# Decoding the Roles of Astrocytes and Hedgehog Signaling in Medulloblastoma

**DOI:** 10.3390/curroncol28040267

**Published:** 2021-08-11

**Authors:** Terence Teixeira Duarte, Silvia Aparecida Teixeira, Luis Gonzalez-Reyes, Rui Manuel Reis

**Affiliations:** 1 Molecular Oncology Research Center, Barretos Cancer Hospital, Barretos 14784-400, Brazil; steixeira.hcb@gmail.com (S.A.T.); rui.reis@hcancerbarretos.com.br (R.M.R.); 2 MCBS Department, School of Medicine, City College of New York, New York, NY 10031, USA; legr707@gmail.com; 3 Life and Health Sciences Research Institute (ICVS), School of Medicine, University of Minho, 4710-057 Braga, Portugal; 4 ICVS/3B’s-PT Government Associate Laboratory, 4710-057 Braga, Portugal

**Keywords:** medulloblastoma, tumor progression, tumor microenvironment, tumor-associated astrocytes, hedgehog signaling, tumor-astrocytes cross talk

## Abstract

The molecular evolution of medulloblastoma is more complex than previously imagined, as emerging evidence suggests that multiple interactions between the tumor cells and components of the tumor microenvironment (TME) are important for tumor promotion and progression. The identification of several molecular networks within the TME, which interact with tumoral cells, has provided new clues to understand the tumorigenic roles of many TME components as well as potential therapeutic targets. In this review, we discuss the most recent studies regarding the roles of astrocytes in supporting sonic hedgehog (SHH) subgroup medulloblastoma (MB) and provide an overview of MB progression through SHH expression and signal transduction mechanisms into the complex tumor microenvironment. In addition, we highlight the associations between tumor and stromal cells as possible prognostic markers that could be targeted with new therapeutic strategies.

## 1. Molecular Signatures of Medulloblastoma Tumorigenesis

Medulloblastoma is the most prevalent malignant brain tumor in children [1,2], while it accounts for only 1–2% of adult brain tumors [3,4,5]. Recognized as a biologically heterogeneous disease, the World Health Organization (WHO) considers there to be four molecular subgroups: wingless-activated (WNT), sonic hedgehog-activated (SHH); Group 3; and Group 4 [4,6,7,8]. Recently, the picture became more complex when 12 different medulloblastoma subtypes were described, including two WNT subtypes, four SHH subtypes, three group 3 subtypes, and three group 4 subtypes, with each subgroup being characterized by specific mutations, copy number variations, transcriptomic/methylomic profiles, and clinical outcomes [4,5,9,10,11,12]. For the SHH subgroup MB, germline or somatic mutations and a copy-number variation are the common drivers that affect critical genes involved in SHH signaling, including *PTCH1* (patched 1 homologue), *SUFU* (suppressor of fused homologue), and *SMO* (smoothened), among others [11,13,14]. The most common genetic events, which occur in both pediatric and adult tumors, are loss-of-function, mutations, or deletions in *PTCH1* and *SUFU*, which act as negative regulators of SHH signaling [13,14,15]. Activation of mutations and amplification of *SMO* or *GLI2* (glioma associated oncogene homologue 2) also lead to constitutive activation of the SHH pathway [16,17]. Germline and somatic *TP53* mutations predominantly coincident with *GLI2* amplifications and are found exclusively in children between the ages of 8 and 17 years [18,19,20,21]. Somatic *TERT* (telomerase) promoter hotspot mutations are also associated with the SHH subgroup [22,23]. Mutation of *PTEN* (phosphatase and tensin homolog) is found in more than 5% of human SHH subgroup MB cases and is associated with decreased expression of PTEN mRNA and proteins in the cerebellum [21,24]. In addition, genetically engineered mouse models (GEMMs) carrying mutations/overexpression of those genes have also been developed to study this medulloblastoma subgroup [25,26,27].

## 2. The Tumor Microenvironment and Its Roles in SHH Subgroup MB

Medulloblastoma can also be viewed through the lens of the tumor microenvironment (TME), and its multiple roles in cancer offer an interesting way to identify the critical steps regulating medulloblastoma biology, disease progression, and overall survival [28,29,30,31,32,33,34,35,36,37,38,39,40,41]. In addition to tumor cells, the tumor microenvironment is characterized by diverse cell populations, including stem-like cells and tumor-associated components such as blood vessels [30], immune cells [36,37], neurons, endothelial cells, microglia [38], macrophages [39,40], and astrocytes [29,31,32,33,41]. The communication between these unique collections of cell types is implicated in therapy resistance [42,43,44], immune infiltration, and inflammation [40]. Since tumor-associated cells could be the focus of therapeutic vulnerability, a comprehensive understanding of the interactions between the tumor cells and the tumor-associated components may provide new opportunities for targeted discoveries. In the SHH subgroup MB, recent studies have highlighted that the cellular diversity within tumors has a critical role in supporting the growth of tumor cells and the robustness of cancer [29,31,33,37,39,40,41,45,46]. In MB-prone mice with a *SMO* mutation, the TME contains tumor cell types that exist across a spectrum of differentiation states and tumor-derived cells that express makers for astrocytic and oligodendrocytic precursors [47]. This suggests that even in a tumor with a single pathway-activation mutation, diverse mechanisms may drive tumor growth, demonstrating the need to target multiple pathways simultaneously for therapeutic effectiveness.

## 3. Astrocytes and the Medulloblastoma Microenvironment: The New Player within the Complex Ecosystem 

Due to increasing evidence of an association between wound healing and the development of tumors, recent studies have investigated the complex functions of astrocytes involved in the support of medulloblastoma growth, as these specialized glial cells are involved in the functional recovery of the central nervous system (CNS) [29,32,33,48,49,50,51,52,53,54,55,56,57,58,59,60,61,62,63,64]. Astrocytes are specialized and heterogeneous cells that are essential modulators of local blood flow as well as being involved in the maintenance of homeostasis of extracellular fluids, ions, and transmitters [61,62]. In a healthy CNS, these glial cells participate in synaptic function and plasticity among other dynamic activities that are crucial for the neural circuit and neurological function and behavior [61,62]. In this context, recent studies have identified SHH signaling as an essential regulator of the molecular identity and functional properties of astrocytes [63,64]. Under normal conditions, astrocytes express the components of the SHH pathway, but do not secrete the SHH protein [65,66]. Recent in vivo studies have shown that the SHH pathway is active in astrocytes of the mature forebrain through the SHH transduction system, which includes the receptor PTCH1 as well as GLI transcription factors [66,67]. Others studies have also demonstrated that the SHH protein is mainly produced by neurons in several brain areas, including dopaminergic neurons [68], the Purkinje cells and mossy cells in the hippocampus, but not in astrocytes or oligodendrocytes [69]. In addition, under physiological stress or pathological conditions, it has been reported that astrocytes may be able to produce and become powerful sources of the SHH protein [70,71,72,73].

In the cerebellum, specialized, unipolar astrocytes called Bergmann glia (BG) have been shown to be capable of responding to the Purkinje-derived SHH protein from the postnatal stage through to adulthood [74]. Mice in which *SMO* is postnatally ablated in BG demonstrate reduced proliferation of granule cell precursors (GCP) and increased differentiation accompanied by a loss of SHH activity. In these animals, WNT signaling is ectopically elevated in GCP, suggesting that this pathway is involved in cross-talk with the SHH pathway, which helps to regulate GCP proliferation [74]. 

Astrocyte reactivity (AR) [50,51,52,53,54,55,56,57,61,63], an ubiquitous, complex, and multistage process, is known to be involved in different CNS pathologies, including trauma [51], inflammation [48], stem-cell repair [53], regeneration [54], peripheral metabolic disorders [55], neurodegenerative diseases [52,56], and tumor progression [46,57,59,75,76,77]. In the context of brain metastasis, reactive astrocytes have a dual role: they limit disease progression during the early stages and, later on, foster tumor growth [57]. During tumor progression, reactive astrocytes are key components of the microenvironment, and their function and crosstalk with other components of the TME have been targets of neuro-oncology research [29,33,45,75,76,77]. Astrocytes can act through paracrine secretion of degradative enzymes, cytokines, chemokines, and growth factors [48] and have multiple primary and branching endfeet that interact with tumor cells, facilitating growth, proliferation, survival, and invasion. Recent studies have demonstrated that, in brain tumors, astrocytes secrete cytokines and trophic factors and contribute to tumor growth, metastasis, and resistance to current therapy [53,55]. In primary gliomas and brain metastases, astrocytes establish gap junctions with tumor cells, and these functional connections are regulated by signaling molecules, such as connexin [55]. In response to these non-cell-autonomous stimuli, astrocytes can produce a multitude of molecular signals that can, in turn, influence many different neural and non-neural cell types, including cells involved in innate immune responses [75]. In parasite infections, astrocytes secrete the SHH protein which, in turn, induces the production of GRP78, an endoplasmic reticulum (ER) chaperone from the heat shock protein family [32]. Under ER stress, it is believed that the activation of *GRP78* may increase cell survival through the unfolded protein response and may also protect cells from ER-stress-induced apoptosis by activating *Bcl-2* and inhibiting *Bak*, *Bax*, *Caspase*, and *CHOP* [32]. In fact, astrocytes facilitate the formation of medulloblastoma tumoroids by secreting SHH proteins and generating the astrocyte-derived extracellular matrix [41]. 

The roles of astrocytes in the medulloblastoma microenvironment have been investigated, and studies have demonstrated that astrocytes secrete CD133, a key cancer stem cell marker that is involved in medulloblastoma tumorigenicity and alters gene expression, increasing invasion and adhesion by medulloblastoma cells [60]. Astrocytes can also have a direct influence on brain tumor stem cells that are activated by several ligands, including SHH, which enriches the stem cell population [78]. These interactions are bi-directional, and tumor stem cells can provide signals that affect the surrounding astrocytes [77]. Interestingly, Liu et al. [78] examined the effects of tumor-associated astrocytes (TAA) in regulating the stemness properties of medulloblastoma stem-like cells in disseminate tumors. These authors showed that TAA produces CCL2 (chemokine ligand 2), shaping the inflammation microenvironment through Notch signaling activation [78].

## 4. SHH-Activated Medulloblastoma: The Indispensable Role of Astrocyte-SHH Secretion in Tumor Progression

Under physiological brain conditions, astrocytes in the cerebellum provide important functions that support the proliferation and migration of granule cell precursors [74,79,80,81,82]. During tumor growth and progression, it is believed that these astrocytes play a critical role in supporting medulloblastoma by secreting the mitogen SHH protein into the tumor microenvironment [29,33,41,74]. In addition to the mutational landscape of SHH signaling components promoting medulloblastoma tumorigenesis, an interesting current topic is the contribution of SHH signaling to the initiation and progression of medulloblastoma. The SHH protein has been hypothesized to influence medulloblastoma in a paracrine manner by being secreted to the stroma which, in turn, signals to the tumor [33]. This could be analogous to the reciprocal signaling networks that SHH establishes during embryonic development [83,84] or in the nigro-striatal system [68]. In an autocrine manner, SHH proteins secreted by TEM cells, including astrocytes, activate signaling in the surrounding stroma, which provides a favorable microenvironment for tumor growth [85]. 

Sonic hedgehog signaling is a highly conserved pathway that has been studied intensively to determine its multiple roles in normal development. It regulates processes involved in tissue patterning, proliferation, and differentiation [86,87,88]. Through its canonical pathway, the SHH ligand acts on target cells through the activation of its receptor PTCH1, relieving the inhibition exerted by PTCH1on SMO [89]. Active SMO initiates a complex intracellular cascade that prevents the processing of GLI2 and GLI3, and promotes their dissociation from SUFU, leading to translocation of full-length and active GLI (GLI^A^) into the nucleus, where they bind to transcriptional targets to regulate cellular gene expression [89,90,91]. In general, GLI1 and GLI2 act as transcriptional activators, while GLI3 represses gene transcription. In SHH-producing cells, the SHH protein may act in an autocrine manner or be secreted in a soluble form through the extracellular milieu to act in a paracrine manner on several long-range target cells [68].

The involvement of SHH signaling in medulloblastoma pathogenesis has been studied extensively, and although the link between the SHH signaling pathway and tumorigenesis is heterogeneous, it is known that the aberrant activation of SHH signaling leads to the growth, proliferation, and invasion of tumor cells [45,85,92,93,94,95]. Mouse models of medulloblastoma are generated by engineering mutations or misexpression of the murine forms of genes mutated in human medulloblastoma. In these models, approximately 15% of mice with a heterozygous *PTCH1* mutation develop tumors in their cerebella, resembling SHH group medulloblastoma in humans [25,96,97]. Conditional deletion of *PTCH1* in cerebellar granule cell precursors (*Math1*) caused medulloblastoma formation with 100% penetrance [98,99,100,101]. These tumors express both GFAP and neurofilaments, suggesting that tumors arise from stem cells that are capable of differentiating along neuronal or glial lineages and challenging the hypothesis that there is a unipotent cell of origin for MB. Using a mouse model with Cre recombinase under control of human regulatory sequences for glial fibrillary acidic protein (*hGFAP*) or *Olig2-tva-cre* drivers to conditionally express an activated *SMO* (*SMOM2*) allele, Schuller et al. [95] demonstrated that *hGFAP+* and *Olig2+* multipotent progenitor populations can produce MB, and these cells retain features of primitive GCP. Using a *Math1-Cre/Ptch1fl/fl* mouse model, Liu et al. [29] revealed that astrocytes are enriched in medulloblastoma, where there is abundant expression of SHH mRNA only in the tumor tissue. These authors showed that, in medulloblastoma tissue, only tumor-associated astrocytes express the SHH protein, suggesting that these astrocytes also secrete this ligand. SHH also contributes to proliferation, and these authors showed that it is downregulated in medulloblastoma cells in vitro. When exogenous SHH proteins were added to the cultures, there was an increase in medulloblastoma cell proliferation [29]. This result was further confirmed within an organotypic slice culture, where the activity blockage of SHH with 5E1 treatment reduced the level of medulloblastoma cell proliferation without increasing apoptosis or cell death. Using a mouse model with ablation of GFAP, Liu et al. [78] also showed significant suppression of medulloblastoma growth in vivo by blocking tumor cell proliferation while promoting differentiation. In addition, using a *Math1-Cre/Ptch1^fl/fl^/Nestin-CFP* mouse model, in which medulloblastoma cells gradually increase the level of Nestin, an intermediate filament protein that plays an inhibitory function on GLI3, these authors showed that the SHH protein was able to induce the production of Nestin mRNA in these mice via tumor-associated astrocytes (TAAs) [29,78]. Genetic ablation of TAA dramatically inhibited Nestin expression in medulloblastoma cells, resulting in reduced proliferation and a blockage of tumor growth. These findings revealed that SHH can signal through a pathway that is independent from GLI1, and a novel non-canonical signaling pathway was revealed in neoplastic cells that involves SHH, SMO, and Nestin [29].

Although tumor cells from the above medulloblastoma models can be readily purified and cultured, these cells do not sustain SHH signaling in vitro [102]. Additionally, primary medulloblastoma cells tend to differentiate in vitro, which negates the possibility of passage or preservation of the medulloblastoma cell lines [29]. Using medulloblastoma cells isolated from *Math1-Cre/Ptch1^fl/fl^* mice, it was shown that these cells autonomously cease proliferation and initiate differentiation, and the expression of all SHH pathway target genes significantly declines over time under adherent culturing conditions [41]. However, these authors observed a supportive role of astrocytes, which secrete the SHH ligand, promoting the development of tumoroids that retain tumorigenicity. The blockade of SHH protein secretion or the removal of astrocytes inhibits the formation of these tumoroids, suggesting that SHH signaling from astrocytes plays an important role in supporting tumor-growth [41].

The interplay between the TME and cellular differentiation is another exciting new area of investigation in cancer biology related to SHH subgroup MB. A recent study showed that tumor-derived astrocytes are involved in reprogramming the microenvironment [33]. Using a Mosaic Analysis with Double Markers (MADM) to observe lineage tracing in SHH medulloblastoma mice models, Yao et al. [33] found that astrocytes within the TME transdifferentiated from granule neuron precursors that never differentiate into astrocytes under physiological conditions [33]. These authors also identified the transcriptome profile of these “astrocyte-like” cells and observed that they closely resemble normal astrocytes [33]. Through in vitro culture experiments, these tumor-derived astrocytes (TuAstrocytes) have been shown to exhibit tumor-supporting roles by accelerating the growth of tumor cells through a paracrine effect. Astrocytes promote the growth of tumors by secreting interleukin-4 which, in turn, induces the tumor-associated macrophages to secret insulin-like growth factor 1 [33]. These results indicate the complex relationship between the TME and tumor cells and highlight the intricate TME community that transdifferentiates as well as a multilateral paracrine network that supports the growth of tumor cells [33,76].

## 5. Therapeutic Approaches and the Intratumoral Heterogeneity of SHH Subgroup MB

Genetic alterations in key components of the sonic hedgehog pathway activate constitutive SHH signaling [13,14,96]. Thus, therapeutic strategies for SHH-pathway-dependent cancers primarily aim at inhibiting the components of the SHH pathway, including the SHH ligand itself as well as SMO and GLI proteins [103,104,105,106]. Historically, the standard chemotherapy regimen for medulloblastoma has largely included cisplatin, vincristine, carboplatin, cyclophosphamide, and lomustine. These alkylating agents are very toxic and generate many side effects [103]. The serendipitous discovery of the steroidal alkaloid cyclopamine, which inhibits SMO and suppresses SHH signaling, acts as a therapeutic to enable pharmacological modulation of the SHH signaling pathway [107,108]. In fact, the most successful strategy has been to target SMO with small-molecule compounds, and two FDA-approved drugs use this strategy [109,110]. In medulloblastoma, two SMO inhibitors, sonidegib (LDE225) and vismodegib (GDC-0449), have been used as therapeutics that specifically target this protein [111,112]. Unfortunately, in children, there is greater concern for developmental toxicity, since the SHH pathway is primarily active during development [113]. Additionally, SHH subgroup MB commonly harbors mutations that result in the emergence of resistance to SMO inhibitors and can occur rapidly [114,115]. To avoid and overcome SMO inhibitor resistance, combination therapies will likely be needed, and there is a continuing effort to identify and therapeutically target components other than this oncoprotein [116,117].

Outside of the canonical SHH pathway, recent advances in SHH subgroup MB research have expanded the list of potential biomarkers to involve other molecular targets [118,119,120], and research is now oriented in new directions towards viable active molecular targets in the SHH pathway. Targeting other key pathways together with SHH signaling is a potential strategy, as there is considerable heterogeneity among SHH subgroup MB, suggesting that non-transcriptional mechanisms are also involved in SHH-signaling-mediated tumorigenesis in medulloblastoma [120]. Among the medulloblastoma groups, SHH subgroup MB displays the highest number of associated macrophages (TAMs), which are critical participants in tumor progression and could be potential therapeutic targets [39,40,121]. Margol et al. [39] showed that the expression of inflammation-related genes, including TAM-related genes, *CD163* and *CSF1R*, is greater in SHH subgroup MB than in other MB subgroups, suggesting that combination therapy aimed at the microenvironment in addition to the tumor’s cells may improve and extend current therapeutic options.

## 6. Novel Targets and Therapeutic Opportunities for Medulloblastoma: A Potential Application of Astrocytes-SHH Medulloblastoma Cross-Talk Research

Current understating of the contribution of the TME to the growth of tumor cells has shifted the focus of neuro-oncology research, moving from exclusively targeting tumoral cells to targeting the tumor microenvironment or signals coming from it, as well as the interactions between them [9,12,33,41,45,78,122]. From multiple studies, it has become clear that the interplay between tumor cells and cells of the tumor microenvironment orchestrate events that are critical to tumor progression, and in this way, many cellular and molecular elements of the microenvironment are emerging as attractive targets for therapeutic strategies [29,35,36,41,43,123]. Although GCPs are the most studied cells regarding the origin of SHH MB and have been the focus of the search for targets in the medulloblastoma for some time, protein receptors and peptide factors from other cellular sources that impact SHH subgroup MB have attracted the attention of researchers more recently.

The G protein-coupled receptor (GPCR) family of proteins is widely dysregulated in cancer and yet is underexploited in oncology. Recent studies have shown that GPCRs can play multiple roles in cancer progression, including proliferation, survival, angiogenesis, metastasis, therapy resistance, and immune evasion upon activation by ligands produced by cancer cells or through the multiplicity of cells within the tumor stroma [122]. The mitogenic ciliary functions of G-protein coupled receptor 37-like 1 (GPR37L1) in SHH–SMO signaling are particularly attractive to target cancer via the tumor microenvironment. The GPR37L1, an orphan G-protein-coupled receptor is a selective marker of cerebellar BG astrocytes [123,124], and it specifically colocalizes and interacts with the PTCH1 protein in discrete areas of these Bergmann glia cell membranes in newborn mice [123]. The Bergmann glial cells possess the primary cilia (PC), which are antenna-like organelles required for sensing and transducing extracellular stimuli [125]. These PC are essential for the regulation of several signaling pathways, such as SHH and WNT, and they can promote tumorigenesis in medulloblastoma [125,126]. Primary cilia have been reliably detected in all cells of pre-neoplastic MB in *PTCH1+/−* mice [31]. Thus, the specific detection of primary cilia could be usefully applied for the study of early, pre-neoplastic MB lesion s [126].

**Figure 1:**
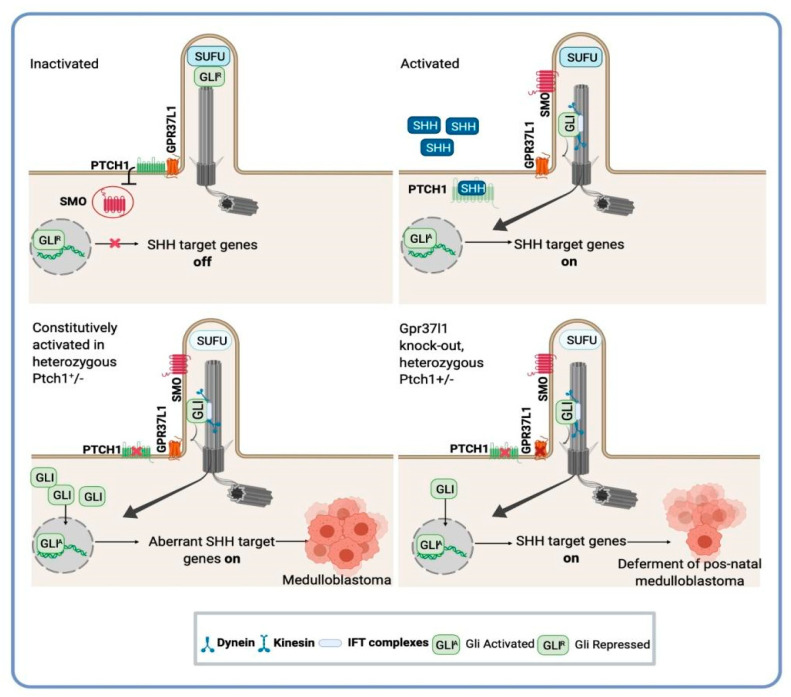
In the absence of the sonic hedgehog ligand (SHH), the negative regulator PTCH1 is present on the ciliary membrane. In this state (Inactivated), suppressor of fused (SUFU) forms a complex with the GLI transcription factors in the periciliary region. SHH binding to PTCH1 (Activated) induces its translocation away from the cilium and promotes the entry of the activating receptor SMO. This process allows the migration of active GLI (GLI^A^) into the nucleus where the transcription of SHH target genes is activated. Mice heterozygous for loss-of-function *PTCH1* mutations have a higher incidence of medulloblastoma [25,96,97]. In a *GPR37l1−/-Ptch1+/−* mouse model, the lack of *GPR37L1* reduced the postnatal tumor occurrence of tumors and decreased the incidence of more aggressive tumor types [31].

*GPR37l1−/−* mice present with precocious Bergmann glia, Purkinje neuron maturation, and increased levels of Purkinje secreted SHH protein, as well as SMO and the intracellular effectors of the SHH-SMO cascade, MYCN and GLI2 [123]. In cerebellar primary astrocyte cultures from *GPR37l1−/−* mouse pups, these astrocytes displayed striking increases in proliferative activity, PTCH1 protein expression and internalization, intracellular cholesterol content, and ciliary localization of SMO, as well as marked production of active SHH signaling [127]. Similar effects were reproduced by treating wild-type astrocytes with a putative prosaptide ligand of the GPR37l1 receptor [127]. Using *GPR37l1−/−Ptch1+/−* mice, Di Pietro et al. (2019) showed that genetic ablation of *GPR37L1* in this medulloblastoma-prone mouse model can reduce the occurrence and severity of postnatal tumors [31] (Figure 1). These authors speculated that this receptor could be involved in the process of BG modulation of SHH production by Purkinje neurons and suggested the involvement of WNT3, a specific inhibitor of SHH-induced neuronal mitogenesis [31]. As GPCRs are the most “druggable” class of proteins currently known, the GPR37L1 receptors have become a highly valuable target for the development of novel therapies, and their use as a specific blocking agent may be an important target for medulloblastoma treatment.

Regarding the critical tumor–stroma interaction related to SHH subgroup MB, Snuderl et al. showed that stromal cells produce Placental growth factor (PlGF), a member of the vascular endothelial growth factor (VEGF) family, which is stimulated via paracrine SHH ligand secretion by the tumor cells [128]. In vitro results have demonstrated that PlGF, its receptor neuropilin 1 (Nrp1), and the MAPK signaling axis are critical for the survival of medulloblastoma cells, and in *Smo/Smo* transgenic mice, the blockade of PlGF with anti-PlGF antibodies was associated with significantly smaller tumors [128]. Together, these findings provide insight into the roles of SHH, PlGF, and Nrp1 in SHH subgroup MB, and as PlGF is dispensable during development, they support the idea that this SHH tumor and PlGF interaction may be used as a therapeutic approach for this pediatric tumor.

Added to the findings highlighted here, the effects of SHH protein secretion by tumor astrocytes on tumor progression and the adaptive transdifferentiation of the tumor and its reliance on astrocytic signals open new perspectives for discovering multiple potential therapeutic targets within the tumor-associated glial cells. With the large amount of genetic and epigenetic data from the subsets of human medulloblastoma, the design of future medulloblastoma therapies must be based on the identification of genes that are specifically expressed in these tumor-associated astrocytes. These genomic data will provide a more comprehensive picture of the molecular scenario regarding the players driving tumor progression and will help to find ways to target those genes, thus increasing the probability of suppressing tumor progression and/or reversing the pro-malignancy effects of SHH secretion by tumor-associated astrocytes.

## Data Availability

Not applicable.

## References

[1] McNeil D.E., Coté T.R., Clegg L., Rorke L.B. (2002). Incidence and trends in pediatric malignancies medulloblastoma/primitive neuroectodermal tumor: A SEER update. Med. Pediatr. Oncol..

[2] Rutkowski S., Cohen B., Finlay J., Luksch R., Ridola V., Valteau-Couanet D., Hara J., Garrè M.L., Grill J. (2010). Medulloblastoma in young children. Pediatr. Blood Cancer.

[3] Northcott P.A., Hielscher T., Dubuc A., Mack S.C., Shih D.J.H., Remke M., Al-Halabi H., Albrecht S., Jabado N., Eberhart C.G. (2011). Pediatric and adult sonic hedgehog medulloblastomas are clinically and molecularly distinct. Acta Neuropathol..

[4] Taylor M.D., Northcott P.A., Korshunov A., Remke M., Cho Y.-J., Clifford S.C., Eberhart C.G., Parsons D.W., Rutkowski S., Gajjar A. (2011). Molecular subgroups of medulloblastoma: The current consensus. Acta Neuropathol..

[5] Hovestadt V., Ayrault O., Swartling F.J., Robinson G.W., Pfister S.M., Northcott P.A. (2019). Medulloblastomics revisited: Biological and clinical insights from thousands of patients. Nat. Rev. Cancer.

[6] Leal L.F., Evangelista A.F., De Paula F.E., Almeida G.C., Carloni A.C., Saggioro F., Stavale J.N., Malheiros S.M., Mançano B., De Oliveira M.A. (2018). Reproducibility of the NanoString 22-gene molecular subgroup assay for improved prognostic prediction of medulloblastoma. Neuropathology.

[7] Pomeroy S.L., Tamayo P., Gaasenbeek M., Sturla L.M., Angelo M., McLaughlin M.E., Kim J.Y.H., Goumnerova L.C., Black P.M., Lau C. (2002). Prediction of central nervous system embryonal tumour outcome based on gene expression. Nature.

[8] Kool M., Korshunov A., Remke M., Jones D.T.W., Schlanstein M., Northcott P.A., Cho Y.-J., Koster J., Meeteren A.S.-V., Van Vuurden D. (2012). Molecular subgroups of medulloblastoma: An international meta-analysis of transcriptome, genetic aberrations, and clinical data of WNT, SHH, Group 3, and Group 4 medulloblastomas. Acta Neuropathol..

[9] Cavalli F.M., Remke M., Rampasek L., Peacock J., Shih D.J.H., Luu B., Garzia L., Torchia J., Nor C., Morrissy S. (2017). Intertumoral Heterogeneity within Medulloblastoma Subgroups. Cancer Cell.

[10] Kumar R., Liu A.P.Y., Northcott P.A. (2019). Medulloblastoma genomics in the modern molecular era. Brain Pathol..

[11] Garcia-Lopez J., Kumar R., Smith K.S., Northcott P.A. (2021). Deconstructing Sonic Hedgehog Medulloblastoma: Molecular Subtypes, Drivers, and Beyond. Trends Genet..

[12] Northcott P.A., Robinson G.W., Kratz C.P., Mabbott D.J., Pomeroy S.L., Clifford S.C., Rutkowski S., Ellison D.W., Malkin D., Taylor M. (2019). Medulloblastoma. Nat. Rev. Dis. Prim..

[13] Raffel C., Jenkins R.B., Frederick L., Hebrink D., Alderete B., Fults D.W., James C.D. (1997). Sporadic medulloblastomas contain PTCH mutations. Cancer Res..

[14] Taylor M.D., Liu L., Raffel C., Hui C.C., Mainprize T.G., Zhang X., Agatep R., Chiappa S., Gao L., Lowrance A. (2002). Mutations in SUFU predispose to medulloblastoma. Nat. Genet..

[15] Brugières L., Pierron G., Chompret A., Paillerets B.B.-D., Di Rocco F., Varlet P., Pierre-Kahn A., Caron O., Grill J., Delattre O. (2009). Incomplete penetrance of the predisposition to medulloblastoma associated with germ-line SUFU mutations. J. Med. Genet..

[16] Gibson P., Tong Y., Robinson G., Thompson M.C., Currle D.S., Eden C., Kranenburg T., Hogg T., Poppleton H., Martin J. (2010). Subtypes of medulloblastoma have distinct developmental origins. Nat. Cell Biol..

[17] Buczkowicz P., Ma J., Hawkins C. (2011). GLI2 Is a Potential Therapeutic Target in Pediatric Medulloblastoma. J. Neuropathol. Exp. Neurol..

[18] Zhukova N., Ramaswamy V., Remke M., Pfaff E., Shih D.J.H., Martin D.C., Castelo-Branco P., Baskin B., Ray P.N., Bouffet E. (2013). Subgroup-Specific Prognostic Implications of TP53 Mutation in Medulloblastoma. J. Clin. Oncol..

[19] Louis D.N., Perry A., Reifenberger G., von Deimling A., Figarella-Branger D., Cavenee W.K., Ohgaki H., Wiestler O.D., Kleihues P., Ellison D.W. (2016). The 2016 World Health Organization Classification of Tumors of the Central Nervous System: A summary. Acta Neuropathol..

[20] Ramaswamy V., Remke M., Bouffet E., Bailey S., Clifford S.C., Doz F., Kool M., Dufour C., Vassal G., Milde T. (2016). Risk stratification of childhood medulloblastoma in the molecular era: The current consensus. Acta Neuropathol..

[21] Da Silva L.S., Mançano B.M., de Paula F.E., dos Reis M.B., de Almeida G.C., Matsushita M., Junior C.A., Evangelista A.F., Saggioro F., Serafini L.N. (2020). Expression of GNAS, TP53, and PTEN Improves the Patient Prognostication in Sonic Hedgehog (SHH) Medulloblastoma Subgroup. J. Mol. Diagn..

[22] Viana-Pereira M., Almeida G.C., Stavale J.N., Malheiro S., Clara C., Lobo P., Pimentel J., Reis R. (2016). Study of hTERT and Histone 3 Mutations in Medulloblastoma. Pathobiology.

[23] Remke M., Ramaswamy V., Peacock J., Shih D.J.H., Koelsche C., Northcott P.A., Hill N., Cavalli F.M.G., Kool M., Wang X. (2013). TERT promoter mutations are highly recurrent in SHH subgroup medulloblastoma. Acta Neuropathol..

[24] Hartmann W., Digon-Söntgerath B., Koch A., Waha A., Endl E., Dani I., Denkhaus D., Goodyer C.G., Sörensen N., Wiestler O.D. (2006). Phosphatidylinositol 3′-Kinase/AKT Signaling Is Activated in Medulloblastoma Cell Proliferation and Is Associated with Reduced Expression ofPTEN. Clin. Cancer Res..

[25] Goodrich L.V., Milenković L., Higgins K.M., Scott M.P. (1997). Altered neural cell fates and medulloblastoma in mouse patched mutants. Science.

[26] Kimura H., Stephen D., Joyner A., Curran T. (2005). Gli1 is important for medulloblastoma formation in Ptc1+/− mice. Oncogene.

[27] Castellino R.C., Barwick B.G., Schniederjan M., Buss M.C., Becher O., Hambardzumyan D., Macdonald T.J., Brat D.J., Durden D.L. (2010). Heterozygosity for Pten promotes tumorigenesis in a mouse model of medulloblastoma. PLoS ONE.

[28] Byrd T., Grossman R.G., Ahmed N. (2012). Medulloblastoma-Biology and microenvironment: A review. Pediatr. Hematol. Oncol..

[29] Liu Y., Yuelling L.W., Wang Y., Du F., Gordon R.E., O'Brien J.A. (2017). Astrocytes promote medulloblastoma progression through hedgehog secretion. Cancer Res..

[30] Hambardzumyan D., Becher O.J., Rosenblum M.K., Pandolfi P.P., Manova-Todorova K., Holland E.C. (2008). PI3K pathway regulates survival of cancer stem cells residing in theperivascular niche following radiation in medulloblastoma in vivo. Genes Dev..

[31] Di Pietro C., La Sala G., Matteoni R., Marazziti D., Tocchini-Valentini G.P. (2019). Genetic ablation of Gpr37l1 delays tumor occurrence in Ptch1 +/− mouse models of medulloblastoma. Exp. Neurol..

[32] Chen K.Y., Chen Y.J., Cheng C.J., Jhan K.Y., Wang L.C. (2020). Excretory/secretory products of Angiostrongyluscantonensis fifth-stage larvae induce endoplasmic reticulum stress via the Sonic hedgehog pathway in mouse astrocytes. Parasit. Vectors.

[33] Yao M., Ventura P.B., Jiang Y., Rodriguez F.J., Wang L., Perry J.S.A. (2020). Astrocytic trans-Differentiation Completes a Multicellular Paracrine Feedback Lop Required for Medulloblastoma Tumor Growth. Cell.

[34] Zhou R., Joshi P., Katsushima K., Liang W., Liu W., Goldenberg N.A. (2020). The emerging field of noncoding RNAs and their importance in pediatric diseases. J. Pediatr..

[35] Chung A.S., Ferrara N. (2010). Targeting the tumor microenvironment with Src kinase inhibition. Clin. Cancer Res..

[36] Binnewies M., Roberts E.W., Kersten K., Chan V., Fearon D.F., Merad M., Coussens L.M., Gabrilovich D.I., Ostrand-Rosenberg S., Hedrick C.C. (2018). Understanding the tumor immune microenvironment (TIME) for effective therapy. Nat. Med..

[37] Gajewski T.F., Schreiber H., Fu Y.-X. (2013). Innate and adaptive immune cells in the tumor microenvironment. Nat. Immunol..

[38] Amarante M.K., Vitiello G.A.F., Rosa M.H., Mancilla I.A., Watanabe M.A.E. (2018). Potential use of CXCL12/CXCR4 and sonic hedgehog pathways as therapeutic targets in medulloblastoma. Acta Oncol..

[39] Margol A.S., Robison N.J., Gnanachandran J., Hung L.T., Kennedy R.J., Vali M., Dhall G., Finlay J.L., Epstein A., Krieger M.D. (2015). Tumor-Associated Macrophages in SHH Subgroup of Medulloblastomas. Clin. Cancer Res..

[40] Pham C.D., Mitchell D.A. (2016). Know your neighbors: Different tumor microenvironments have implications in immunotherapeutic targeting strategies across MB subgroups. Oncoimmunology.

[41] Cheng Y., Franco-Barraza J., Wang Y., Zheng C., Zhang L., Qu Y., Long Y., Cukierman E., Yang Z.-J. (2020). Sustained hedgehog signaling in medulloblastoma tumoroids is attributed to stromal astrocytes and astrocyte-derived extracellular matrix. Lab. Investig..

[42] Raviraj R., Nagaraja S.S., Selvakumar I., Mohan S., Nagarajan D. (2020). The epigenetics of brain tumors and its modulation during radiation: A review. Life Sci..

[43] Hirata E., Sahai E. (2017). Tumor microenvironment and differential responses to therapy. Cold Spring Harb. Perspect. Med..

[44] Hanahan D., Weinberg R.A. (2011). Hallmarks of Cancer: The Next Generation. Cell.

[45] Tamayo-Orrego L., Charron F. (2019). Recent advances in SHH medulloblastoma progression: Tumor suppressor mechanisms and the tumor microenvironment. F1000Research.

[46] Raza M., Prasad P., Gupta P., Kumar N., Sharma T., Rana M., Goldman A., Sehrawat S. (2018). Perspectives on the role of brain cellular players in cancer-associated brain metastasis: Translational approach to understand molecular mechanism of tumor progression. Cancer Metastasis Rev..

[47] Ocasio J., Babcock B., Malawsky D., Weir S.J., Loo L., Simon J.M., Zylka M.J., Hwang D., Dismuke T., Sokolsky M. (2019). scRNA-seq in medulloblastoma shows cellular heterogeneity and lineage expansion support resistance to SHH inhibitor therapy. Nat. Commun..

[48] Sofroniew M.V. (2014). Multiple roles for astrocytes as effectors of cytokines and inflammatory mediators. Neuroscientist.

[49] Khakh B.S., Sofroniew M.V. (2015). Diversity of astrocyte functions and phenotypes in neural circuits. Nat. Neurosci..

[50] Anderson M.A., Ao Y., Sofroniew M.V. (2014). Heterogeneity of reactive astrocytes. Neurosci. Lett..

[51] Burda J.E., Bernstein A.M., Sofroniew M.V. (2016). Astrocyte roles in traumatic brain injury. Exp. Neurol..

[52] Tong X., Ao Y., Faas G.C., Nwaobi S.E., Xu J., Haustein M.D., Anderson M.A., Mody I., Olsen M., Sofroniew M.V. (2014). Astrocyte Kir4.1 ion channel deficits contribute to neuronal dysfunction in Huntington’s disease model mice. Nat. Neurosci..

[53] Robel S., Berninger B., Götz M. (2011). The stem cell potential of glia: Lessons from reactive gliosis. Nat. Rev. Neurosci..

[54] Silver J., Miller J.H. (2004). Regeneration beyond the glial scar. Nat. Rev. Neurosci..

[55] Zamanian J.L., Xu L., Foo L.C., Nouri N., Zhou L., Giffard R.G., Barres B.A. (2012). Genomic analysis of reactive astrogliosis. J. Neurosci..

[56] Pekny M., Pekna M. (2016). Reactive gliosis in the pathogenesis of CNS diseases. Biochim. Biophys Acta..

[57] Wasilewski D., Priego N., Fustero-Torre C., Valiente M. (2017). Reactive astrocytes in brain metastasis. Front. Oncol..

[58] Placone A.L., Quiñones-Hinojosa A., Searson P.C. (2016). The role of astrocytes in the progression of brain cancer: Complicating the picture of the tumor microenvironment. Tumor Biol..

[59] Brandao M., Simon T., Critchley G., Giamas G. (2019). Astrocytes, the rising stars of the glioblastoma microenvironment. Glia.

[60] Gronseth E., Gupta A., Koceja C., Kumar S., Kutty R.G., Rarick K., Wang L., Ramchandran R. (2020). Astrocytes influence medulloblastoma phenotypes and CD133 surface expression. PLoS ONE.

[61] Nedergaard M., Ransom B., Goldman S.A. (2003). New roles for astrocytes: Redefining the functional architecture of the brain. Trends Neurosci..

[62] Barres B.A. (2008). The Mystery and Magic of Glia: A perspective on their roles in health and disease. Neuron.

[63] Sofroniew M.V. (2009). Molecular dissection of reactive astrogliosis and glial scar formation. Trends Neurosci..

[64] Sofroniew M.V. (2020). Astrocyte reactivity: Subtypes, states, and functions in cns innate immunity. Trends Immunol..

[65] Traiffort E., Charytoniuk D., Watroba L., Faure H., Sales N., Ruat M. (1999). Discrete localizations of hedgehog signalling components in the developing and adult rat nervous system. Eur. J. Neurosci..

[66] Garcia A.D., Petrova R., Eng L., Joyner A.L. (2010). Sonic Hedgehog regulates discrete populations of astrocytes in the adult muse forebrain. J. Neurosci..

[67] Jiao J., Chen D.F. (2008). Induction of neurogenesis in nonconventional neurogenic regions of the adult central nervous systeby niche astrocyte-produced signals. Stem Cells.

[68] Gonzalez-Reyes L.E., Verbitsky M., Blesa J., Jackson-Lewis V., Paredes D., Tillack K., Phani S., Kramer E., Przedborski S., Kottmann A. (2012). Sonic Hedgehog Maintains Cellular and Neurochemical Homeostasis in the Adult Nigrostriatal Circuit. Neuron.

[69] Gonzalez-Reyes L.E., Chiang C.-C., Zhang M., Johnson J., Arrillaga-Tamez M., Couturier N.H., Reddy N., Starikov L., Capadona J.R., Kottmann A.H. (2019). Sonic Hedgehog is expressed by hilar mossy cells and regulates cellular survival and neurogenesis in the adult hippocampus. Sci. Rep..

[70] Amankulor N.M., Hambardzumyan D., Pyonteck S.M., Becher O.J., Joyce J.A., Holland E.C. (2009). Sonic hedgehog pathway activation is induced by acute brain injury and regulated by injury-related inflammation. J. Neurosci..

[71] Alvarez J.I., Dodelet-Devillers A., Kebir H., Ifergan I., Fabre P.J., Terouz S., Sabbagh M., Wosik K., Bourbonnière L., Bernard M. (2011). The Hedgehog Pathway Promotes Blood-Brain Barrier Integrity and CNS Immune Quiescence. Science.

[72] Sirko S., Behrendt G., Johansson P., Tripathi P., Costa M., Bek S., Heinrich C., Tiedt S., Colak D., Dichgans M. (2013). Reactive Glia in the Injured Brain Acquire Stem Cell Properties in Response to Sonic Hedgehog. Cell Stem Cell.

[73] Pitter K., Tamagno I., Feng X., Ghosal K., Amankulor N., Holland E.C., Hambardzumyan D. (2014). The SHH/Gli pathway is reactivated in reactive glia and drives proliferation in response to neurodegeneration-induced lesions. Glia.

[74] Cheng F.Y., Fleming J.T., Chiang C. (2018). Bergmann glial Sonic hedgehog signaling activity is required for proper cerebellar cortical expansion and architecture. Dev. Biol..

[75] Priego N., Valiente M. (2019). The potential of astrocytes as immune modulators in brain tumors. Front. Immunol..

[76] Zhang G., Rich J.N. (2020). Reprogramming the microenvironment: Tricks of tumor-derived astrocytes. Cell Res..

[77] Gronseth E., Wang L., Harder D.R., Ramchandran R. (2018). The Role of Astrocytes in Tumor Growth and Progression. Astrocyte-Physiology and Pathology.

[78] Liu H., Sun Y., O’Brien J., Franco-Barraza J., Qi X., Yuan H., Jin W., Zhang J., Gu C., Zhao Z. (2020). Necroptotic astrocytes contribute to maintaining stemness of disseminated medulloblastoma through CCL2 secretion. Neuro. Oncol..

[79] Shiga T., Ichikawa M., Hirata Y. (1983). Spatial and temporal pattern of postnatal proliferation of Bergmann glial cells in rat cerebellum: An autoradiographic study. Anat. Embryol..

[80] Buffo A., Rossi F. (2013). Origin, lineage and function of cerebellar glia. Prog. Neurobiol..

[81] Terry T.T., Cheng T., Mahjoub M., Zong H. (2020). Mosaic Analysis with Double Markers reveals IGF1R function in granule cell progenitors during cerebellar development. Dev. Biol..

[82] Araujo A.P.B., Carpi-Santos R., Gomes F.C.A. (2019). The role of astrocytes in the development of the cerebellum. Cerebellum.

[83] Wallace V.A. (1999). Purkinje-cell-derived Sonic hedgehog regulates granule neuron precursor cell proliferation in the developing mouse cerebellum. Curr. Biol..

[84] Wechsler-Reya R.J., Scott M.P. (1999). Control of neuronal precursor proliferation in the cerebellum by sonic hedgehog. Neuron.

[85] Skoda A.M., Simovic D., Karin V., Kardum V., Vranic S., Serman L. (2018). The role of the hedgehog signaling pathway in cancer: A comprehensive review. Bosn. J. Basic Med. Sci..

[86] Ingham P.W., McMahon A.P. (2001). Hedgehog signaling in animal development: Paradigms and principles. Genes Dev..

[87] McMahon A.P., Ingham P.W., Tabin C.J. (2003). Developmental roles and clinical significance of Hedgehog signaling. Curr. Top. Dev. Biol..

[88] Briscoe J., Thérond P.P. (2013). The mechanisms of Hedgehog signalling and its roles in development and disease. Nat. Rev. Mol. Cell Biol..

[89] Taipale J., Cooper M.K., Maiti T., Beachy P.A. (2002). Patched acts catalytically to suppress the activity of smoothened. Nature.

[90] Rohatgi R., Milenkovic L., Scott M.P. (2007). Patched1 regulates hedgehog signaling at the primary cilium. Science.

[91] Varjosalo M., Taipale J. (2008). Hedgehog: Functions and mechanisms. Genes Dev..

[92] Oliver T., Read T.-A., Kessler J.D., Mehmeti A., Wells J.F., Huynh T.T.T., Lin S.M., Wechsler-Reya R.J. (2005). Loss of patched and disruption of granule cell development in a pre-neoplastic stage of medulloblastoma. Development.

[93] Romer J., Curran T. (2005). Targeting medulloblastoma: Small-molecule inhibitors of the Sonic Hedgehog pathway as potential cancer therapeutics. Cancer Res..

[94] Merk D.J., Segal R.A. (2018). Sonic hedgehog signaling is blue: Insights from the patched mutant mice. Trends Neurosci..

[95] Schüller U., Heine V., Mao J., Kho A.T., Dillon A.K., Han Y.-G., Huillard E., Sun T., Ligon A.H., Qian Y. (2008). Acquisition of Granule Neuron Precursor Identity Is a Critical Determinant of Progenitor Cell Competence to Form Shh-Induced Medulloblastoma. Cancer Cell.

[96] Zurawel R.H., Allen C., Wechsler-Reya R., Scott M.P., Raffel C. (2000). Evidence that haploinsufficiency of Ptch leads to medulloblastoma in mice. Genes Chromosomes Cancer.

[97] Mao J., Ligon K.L., Rakhlin E.Y., Thayer S.P., Bronson R.T., Rowitch D., McMahon A.P. (2006). A novel somatic mouse model to survey tumorigenic potential applied to the Hedgehog pathway. Cancer Res..

[98] YYang Z.-J., Ellis T., Markant S.L., Read T.-A., Kessler J.D., Bourboulas M., Schüller U., Machold R., Fishell G., Rowitch D. (2008). Medulloblastoma can be initiated by deletion of Patched in lineage-restricted progenitors or stem cells. Cancer Cell.

[99] Schuler D. (2010). A kemoterápiaszerepe a gyermekkori medulloblastoma kezelésében. [The role of chemotherapy in pediatric medulloblastoma]. Magy. Onkol..

[100] Ayrault O., Zhao H., Zindy F., Qu C., Sherr C.J., Roussel M.F. (2010). Atoh1 inhibits neuronal differentiation and collaborates with Gli1 to generate medulloblastoma-initiating cells. Cancer Res..

[101] Pazzaglia S., Tanori M., Mancuso M., Gessi M., Pasquali E., Leonardi S., Oliva M.A., Rebessi S., Di Majo V., Covelli V. (2006). Two-hit model for progression of medulloblastoma preneoplasia in Patched heterozygous mice. Oncogene.

[102] Sasai K., Romer J.T., Lee Y., Finkelstein D., Fuller C., McKinnon P.J., Curran T. (2006). Shh pathway activity is down-regulated in cultured medulloblastoma cells: Implications for preclinical studies. Cancer Res..

[103] Rimkus T.K., Carpenter R.L., Qasem S., Chan M., Lo H.W. (2016). Targeting the sonic hedgehog signaling pathway: Review of smoothened and GLI inhibitors. Cancers.

[104] Wu F., Zhang Y., Sun B., McMahon A.P., Wang Y. (2017). Hedgehog Signaling: From basic biology to cancer therapy. Cell Chem. Biol..

[105] Severini L.L., Quaglio D., Basili I., Ghirga F., Bufalieri F., Caimano M., Balducci S., Moretti M., Romeo I., Loricchio E. (2019). A Smo/Gli Multitarget Hedgehog Pathway Inhibitor Impairs Tumor Growth. Cancers.

[106] Packer R.J., Gajjar A., Vezina G., Rorke-Adams L., Burger P.C., Robertson P.L., Bayer L., LaFond D., Donahue B.R., Marymont M.H. (2006). Phase III Study of Craniospinal Radiation Therapy Followed by Adjuvant Chemotherapy for Newly Diagnosed Average-Risk Medulloblastoma. J. Clin. Oncol..

[107] Cooper M.K., Porter J.A., Young K.E., Beachy P.A. (1998). Teratogen-mediated inhibition of target tissue response to Shh signaling. Science.

[108] Incardona J.P., Gaffield W., Kapur R.P., Roelink H. (1998). The teratogenic Veratrum alkaloid cyclopamine inhibits sonic hedgehog signal transduction. Development.

[109] Lou E., Nelson A.C., Kool M. (2019). Differential response of SHH-expressing adult medulloblastomas to the sonic hedgehog inhibitor vismodegib: Whole-genome analysis. Cancer Biol. Ther..

[110] Wu T., Qu P.-R., Zhang S., Li S.-W., Zhang J., Wang B., Liu P., Li C.-D., Zhao F. (2020). The clinical treatment and outcome of cerebellopontine angle medulloblastoma: A retrospective study of 15 cases. Sci. Rep..

[111] Kieran M.W., Chisholm J., Casanova M., Brandes A.A., Aerts I., Bouffet E., Bailey S., Leary S., Macdonald T.J., Mechinaud F. (2017). Phase I study of oral sonidegib (LDE225) in pediatric brain and solid tumors and a phase II study in children and adults with relapsed medulloblastoma. Neuro-Oncology.

[112] Lorusso P.M., Rudin C., Reddy J.C., Tibes R., Weiss G.J., Borad M.J., Hann C.L., Brahmer J.R., Chang I., Darbonne W.C. (2011). Phase I Trial of Hedgehog Pathway Inhibitor Vismodegib (GDC-0449) in Patients with Refractory, Locally Advanced or Metastatic Solid Tumors. Clin. Cancer Res..

[113] Li Y., Song Q., Day B.W. (2019). Phase I and phase II sonidegib and vismodegib clinical trials for the treatment of paediatric and adult MB patients: A systemic review and meta-analysis. Acta Neuropathol. Commun..

[114] Atwood S., Sarin K., Whitson R.J., Li J.R., Kim G., Rezaee M., Ally M.S., Kim J., Yao C., Chang A.L.S. (2015). Smoothened Variants Explain the Majority of Drug Resistance in Basal Cell Carcinoma. Cancer Cell.

[115] Danial C., Sarin K.Y., Oro A.E., Chang A.L. (2016). An investigator-initiated open-label trial of sonidegib in advanced basal cell carcinoma patients resistant to vismodegib. Clin. Cancer Res..

[116] Sharpe H.J., Wang W., Hannoush R.N., de Sauvage F.J. (2015). Regulation of the oncoprotein Smoothened by small molecules. Nat. Chem. Biol..

[117] Li K.K., Lau K.M., Ng H.K. (2013). Signaling pathway and molecular subgroups of medulloblastoma. Int. J. Clin. Exp. Pathol..

[118] Xin M., Ji X., De La Cruz L.K., Thareja S., Wang B. (2018). Strategies to target the Hedgehog signaling pathway for cancer therapy. Med. Res. Rev..

[119] Mille F., Tamayo-Orrego L., Lévesque M., Remke M., Korshunov A., Cardin J., Bouchard N., Izzi L., Kool M., Northcott P.A. (2014). The Shh Receptor Boc Promotes Progression of Early Medulloblastoma to Advanced Tumors. Dev. Cell.

[120] Thompson E.M., Ashley D., Landi D. (2020). Current medulloblastoma subgroup specific clinical trials. Transl. Pediatr..

[121] Maximov V., Chen Z., Wei Y., Robinson M.H., Herting C.J., Shanmugam N., Rudneva V.A., Goldsmith K.C., Macdonald T.J., Northcott P.A. (2019). Tumour-associated macrophages exhibit anti-tumoural properties in Sonic Hedgehog medulloblastoma. Nat. Commun..

[122] Wu V., Yeerna H., Nohata N., Chiou J., Harismendy O., Raimondi F., Inoue A., Russell R.B., Tamayo P., Gutkind J.S. (2019). Illuminating the Onco-GPCRome: Novel G protein–coupled receptor-driven oncocrine networks and targets for cancer immunotherapy. J. Biol. Chem..

[123] DI Pietro C., Marazziti D., Lasala G., Abbaszadeh Z., Golini E., Matteoni R., Tocchini-Valentini G.P. (2017). Primary Cilia in the Murine Cerebellum and in Mutant Models of Medulloblastoma. Cell. Mol. Neurobiol..

[124] Marazziti D., Di Pietro C., Golini E., Mandillo S., Lasala G., Matteoni R., Tocchini-Valentini G.P. (2013). Precocious cerebellum development and improved motor functions in mice lacking the astrocyte cilium-, patched 1-associated Gpr37l1 receptor. Proc. Natl. Acad. Sci. USA.

[125] Han Y.G., Kim H.J., Dlugosz A.A., Ellison D.W., Gilbertson R.J., Alvarez-Buylla A. (2009). Dual and opposing roles of primary cilia in medulloblastoma development. Nat. Med..

[126] Han Y.G., Alvarez-Buylla A. (2010). Role of primary cilia in brain development and cancer. Curr. Opin. Neurobiol..

[127] La Sala G., Di Pietro C., Matteoni R., Bolasco G., Marazziti D., Tocchini-Valentini G.P. (2021). Gpr37l1/prosaposin receptor regulates Ptch1 trafficking, Shh production, and cell proliferation in cerebellar primary astrocytes. J. Neurosci. Res..

[128] Snuderl M., Batista A., Kirkpatrick N.D., de Almodovar C.R., Riedemann L., Walsh E.C., Anolik R., Huang Y., Martin J., Kamoun W. (2013). Targeting placental growth factor/neuropilin 1 pathway inhibits growth and spread of medulloblastoma. Cell.

